# A Single-Cell Gene-Expression Profile Reveals Inter-Cellular Heterogeneity within Human Monocyte Subsets

**DOI:** 10.1371/journal.pone.0144351

**Published:** 2015-12-09

**Authors:** Susanne T. Gren, Thomas B. Rasmussen, Sabina Janciauskiene, Katarina Håkansson, Jens G. Gerwien, Olof Grip

**Affiliations:** 1 Cellular Pharmacology, Novo Nordisk A/S, Måløv, Denmark; 2 Department of Clinical Sciences Malmö, Lund University, Malmö, Sweden; 3 Clinical Immunology, Copenhagen University Hospital, Copenhagen, Denmark; 4 Department of Respiratory Medicine, Hannover Medical School, Hannover, Germany; 5 Global Biobanking Management, Novo Nordisk A/S, Måløv, Denmark; Massachusetts General Hospital, UNITED STATES

## Abstract

Human monocytes are a heterogeneous cell population classified into three different subsets: Classical CD14^++^CD16^-^, intermediate CD14^++^CD16^+^, and non-classical CD14^+^CD16^++^ monocytes. These subsets are distinguished by their differential expression of CD14 and CD16, and unique gene expression profile. So far, the variation in inter-cellular gene expression within the monocyte subsets is largely unknown. In this study, the cellular variation within each human monocyte subset from a single healthy donor was described by using a novel single-cell PCR gene-expression analysis tool. We investigated 86 different genes mainly encoding cell surface markers, and proteins involved in immune regulation. Within the three human monocyte subsets, our descriptive findings show multimodal expression of key immune response genes, such as *CD40*, *NFⱪB1*, *RELA*, *TLR4*, *TLR8* and *TLR9*. Furthermore, we discovered one subgroup of cells within the classical monocytes, which showed alterations of 22 genes e.g. *IRF8*, *CD40*, *CSF1R*, *NFⱪB1*, *RELA and TNF*. Additionally one subgroup within the intermediate and non-classical monocytes also displayed distinct gene signatures by altered expression of 8 and 6 genes, respectively. Hence the three monocyte subsets can be further subdivided according to activation status and differentiation, independently of the traditional classification based on cell surface markers. Demonstrating the use and the ability to discover cell heterogeneity within defined populations of human monocytes is of great importance, and can be useful in unravelling inter-cellular variation in leukocyte populations, identifying subpopulations involved in disease pathogenesis and help tailor new therapies.

## Introduction

Blood monocytes are a heterogeneous population of innate immune leukocytes. They are involved in the innate immune response to pathogens by phagocytosis, the release of reactive oxygen species, cytokines and chemokines and by antigen presentation, thereby modulating and activating cells within the adaptive immune system [1]. The diversity within the human blood monocyte subpopulations has become evident in recent years. Based on the differential expression of the co-receptor to lipopolysaccharide (LPS) CD14 and the Fcγ receptor (FcγR)-III CD16, human monocytes can be divided into different subpopulations [2]. First, two subpopulations were recognized, namely the CD14^+^CD16^-^ and the CD14^-^CD16^+^ monocytes [3], that were shown to have distinct biological functions [4] and a proportional increase of the CD14^-^CD16^+^ monocyte subset were seen in a variety of chronic and inflammatory diseases [5–8]. Thus, later it became evident that the CD16^+^ monocytes could be further divided into two subsets according to the level of CD14 expression. Three monocyte subpopulations have now been identified and characterized in humans [9], whereas two subsets are identified according to the expression of GR1 and Ly6C in mice [2]. The human monocytes have been given the following notation: Classical (CD14^++^CD16^-^), Intermediate (CD14^++^CD16^+^) and Non-classical (CD14^+^CD16^++^) monocytes [10]. Classical and intermediate monocytes are shown to be homologs to the mouse Gr^+^Ly6C^+^, whereas the non-classical monocytes resemble the mouse Gr^-^Ly6C^-^ monocytes [9]. The heterogeneity within monocytes has been unravelled by the expression of cell surface markers and by using gene expression profiling. Human classical monocytes express a diversity of genes that favours their involvement in migration, bacterial sensing, phagocytosis, immune responses and many pro-inflammatory genes, which support their role in inflammation. In contrast, intermediate monocytes display genes that account for a profile that is more prone to antigen-presenting [11] whereas genes up-regulated in non-classical monocytes are mainly involved in patrolling, sensing of nucleic acids and viruses [9].

Several studies have implied that LPS-stimulated intermediate and non-classical monocytes are the most pro-inflammatory among the three subsets [9,11]. We have previously shown that the classical monocytes are the most pro-inflammatory in regard to cytokine secretion and MMP release when stimulated with LPS and immune complexes [12]. This is in agreement with the high expression of CD14 and the FcγRI CD64 [11,12]. In relation to disease pathogenesis, the subdivision of CD16^+^ monocytes showed that in chronic and autoimmune diseases, for example Crohn’s disease (CD), the intermediate monocytes were expanded in the peripheral blood in patients with active inflammation [7,12–14], whereas the classical subset was decreased [12]. However, the role of the classical, intermediate and non-classical human monocytes in health and disease has not been fully elucidated.

Previous gene-expression profiling has distinguished the three monocyte subsets and addressed mechanisms of transcriptional regulation and differential functional genes [11,15,16]. However, an increasing body of evidence indicates that cell subpopulations can be comprised of cells with distinct gene expression profiles, though this is masked when using techniques such as micro-array analysis. To the best of our knowledge, no studies have performed single-cell gene expression analysis of the three monocyte subsets, a technique that can elucidate the inter-cellular heterogeneity. Here, we investigated the three monocyte subsets using the single-cell gene analysis technique from Fluidigm. We investigated 86 selected genes, involved mainly in adhesion, migration, phagocytosis, tissue remodelling and immune functionality, in order to obtain a greater knowledge of the heterogeneity within each monocyte subset. Our results showed a hitherto unknown inter-cellular variation within the three human monocyte subsets, which other techniques such as micro-array analysis and flow cytometry are unable to identify.

## Results

### Gene expression profile

**Fig 1:**
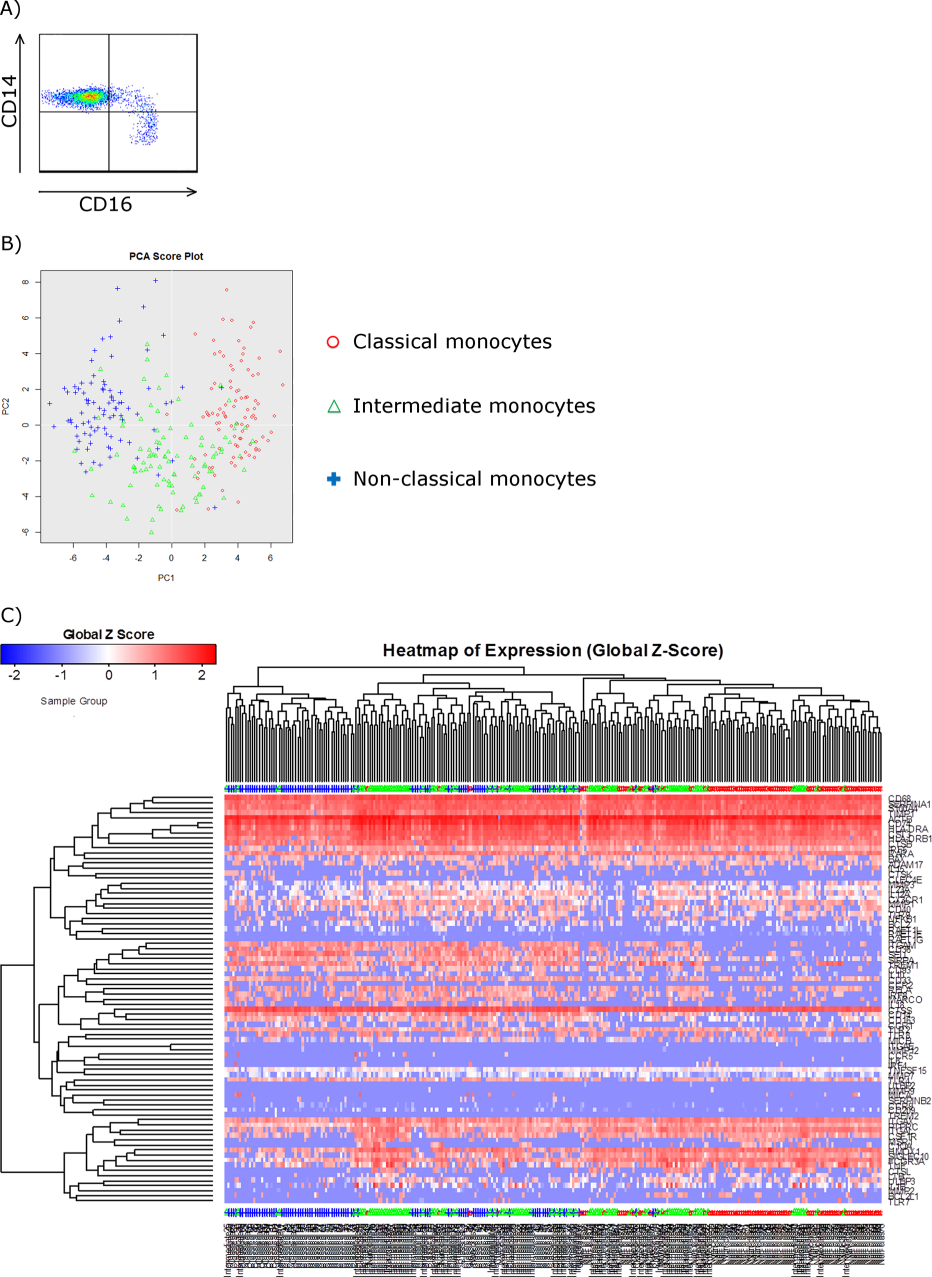
Single-cell gene expression analysis on human monocytes. Human monocytes were single-cell sorted according to the expression of the cell surface markers CD14 and CD16 and gene expression on single cells was assessed. A) Representative plot of flow cytometry analysis demonstrating the monocyte subset gating strategy, used to single-cell sort monocytes from the three classified monocyte subsets (*n =* 1). Classical (CD14^++^CD16^-^) monocytes, intermediate (CD14^++^CD16^+^) monocytes and non-classical (CD14^++^CD16^+^) monocytes are depicted in the upper left quadrant, upper right quadrant and lower right quadrant, respectively. B) Principal component analyses (PCA) of single-cell PCR gene expression analysis data showing genetic clustering of the three monocyte subsets. The PCA plot confirmed the classification of the three human monocyte subsets done by flow cytometry, visualized by gene families. Each dot represents a single cell. C) Heatmap of gene expression values for PCA showing hierarchical clustering of single-cell PCR gene expression data from the three human monocyte sub-populations. The analysis revealed cellular heterogeneity by distinct gene signatures. Red circles = classical monocytes (*n* = 94 cells), green triangles = intermediate monocytes (*n* = 92 cells), and blue pluses = non-classical monocytes (*n* = 80 cells).

Live, single cells within the three human monocyte subsets were single-cell-sorted according to forward and side scatter, and the expression of the cellular surface markers CD14 and CD16 (Fig 1A). Cellular lysates from 94 classical monocytes, 92 intermediate monocytes and 90 non-classical monocytes were converted to cDNA and amplified with 85 target genes (Table 1). The 85 genes analysed were chosen based on differential expression shown in earlier micro-array results [11,15], and by their relation to cell function, as well as biological or immune regulated processes. The genes were plotted into a PCA score plot showing that the single-sorted cells were clustered according to their subset, classical, intermediate or non-classical, verifying that the cells were sorted correctly (Fig 1B). The expression of each gene was analysed using the SINGuLAR R package. No signal in the Fluidigm qPCR were interpreted as expression below detection limit and referred to as non-expression. We found a differential expression of 80 genes within the 3 subgroups of monocytes (Fig 1C and Table 2). Five of the genes, CCR7, MMP12, MRC1, ULBP1 and ULBP2 were not expressed by any of the subsets. Four genes, CCR2, CD163, CLEC4E, and SERPINB2 were exclusively not expressed by the non-classical subset. Expression of IL6 was not detectable in the classical subset and very low expression was observed in the intermediate and non-classical subset. MMP9 was weakly expressed by the classical and non-classical monocytes and no expression was found in the intermediate subset. TLR3 was not expressed by the classical and intermediate, but weakly by the non-classical monocytes, whereas TREM2 was weakly expressed by the classical subset but not by the intermediate and non-classical subsets. Expression of CCR5 and RAET1G was found only in the intermediate monocytes. These data show that there is great diversity within the three monocyte subsets and that the subsets have a differential gene expression profile with regard to the genes investigated in this study.

**Table 1:** Genes of interest investigated in the three monocyte subsets.

Gene symbol	Gene title	Protein function
ACTB	Actin beta	Conserved motif, ubiquitously expressed in all eukaryotic cells
ADAM17	ADAM Metallopeptidase Domain 17	Cleave membrane-bound precursor TNF-α
BAX	BCL2-associated X protein	Apoptotic activator
BCL2	B-cell lymphoma 2	Apoptotic suppressor
BCL2L1	BCL2-like1	Inhibits activation of caspases
C1QA	Complement component, q subcomponent, A chain	First component of complement system
CCR1	Chemokine (C-C motif) receptor 1	Recruitment of effector cells to sites of inflammation
CCR2	Chemokine (C-C motif) receptor 2	Mediates monocyte chemotaxis
CCR5	Chemokine (C-C motif) receptor 5	Receptor for a number of inflammatory CC-chemokines
CCR7	Chemokine (C-C motif) receptor 7	Migration of memory T cells and stimulate DC maturation
CCR9	Chemokine (C-C motif) receptor 9	thymocyte recruitment and development, localization to the gastrointestinal tract
CD14	CD14 molecule	Innate immune responses to bacterial lipopolysaccharide
CD163	CD163 molecule	Phagocytosis of hemoglobin/haptoglobin complexes
CD209	CD209 molecule	C-type lectin, pathogen recognition receptor and cell adhesion
CD33	CD33 molecule	Sialic acid adhesion molecule
CD36	CD36 molecule	Receptor for thrombospondin, function in cell adhesion
CD40	CD40 molecule	Receptor for CD40L, co-stimulatory molecule, T and B cell activation
CD68	CD68 molecule	Intracellular lysosomal metabolism and extracellular cell-cell and cell-pathogen interaction
CD74	CD74 molecule	Invariant chain, regulates antigen presentation
CD93	CD93 molecule	Intercellular adhesion and clearance of apoptotic cells
CLEC4E	C-type lectin domain family 4, member E	Recognise pathogenic fungi
CSF1R	Colony stimulating factor 1 receptor	Regulates survival, proliferation and differentiation of hematopoietic precursor cells, promote release of inflammatory cytokines in response to CSF1
CST3	Cystatin C	Inhibitor of cysteine proteases
CTSB	Cathepsin B	Intracellular degradation and turnover of proteins
CTSK	Cathepsin K	Bone remodelling, endoprotease activity against fibrinogen
CTSL	Cathepsin L	Degradation of proteins in lysosomes
CTSS	Cathepsin S	Degradation and turnover of proteins, maturation MHC II complex
CX3CR1	Chemokine (C-X3-C motif) receptor 1	Receptor for fraktalkine, mediates adhesion and migration
FCGR3A	Fc gamma receptor IIIA	Receptor for Fc region of immunoglobulin G
HLA-DRA	Major histocompatibility complex, class II, DR alpha	Alpha chain of HLA-DR class II complex, antigen presentation
HLA-DRB1	Major histocompatibility complex, class II, DR beta	Beta chain of HLA-DR class II complex, antigen presentation
HMOX1	Heme oxygenase (Decycling) 1	Cleave the heme ring, to form biliverdin
IL10	Interleukin 10	Immune regulation, inhibits number of cytokines, enhances cell survival and proliferation
IL12A	Interleukin 12A	Act as growth factor for T cells and NK cells, stimulate production of IFN-gamma by T cells
IL15	Interleukin 15	Stimulate proliferation of T cells
IL18	Interleukin 18	Stimulate IFN-gamma production by T cells, augments NK cell activity
IL1B	Interleukin 1beta	Inflammatory mediator, cell proliferation, differentiation, and apoptosis
IL23A	Interleukin 23A	Stimulate memory T cells, stimulates production of IFN-gamma active response to infection
IL6	Interleukin 6	Induce acute phase response. Leukocyte differentiation
IRF4	Interferon regulatory factor 4	Lymphocyte specific transcriptional activator that regulates TLR signalling
IRF5	Interferon regulatory factor 5	Transcriptional activator that regulates TLR7 and TLR9 signalling
IRF8	Interferon regulatory factor 8	Binds to the upstream region of type I IFN and IFN-inducible MHC class I genes
ITGAE	Integrin Alpha E (CD103 molecule)	Intergrin alpha E/beta-7 is a receptor for E-cadherin
ITGAL	Integrin Alpha L (Antigen CD11A)	Intercellular adhesion through interaction with ICAM1-3, leukocyte-endothelial cell interaction, antibody dependent killing by monocytes and granulocytes
ITGAM	Integrin Alpha M (CD11B)	Adherence to stimulated endothelium, receptor for iC3B fragtment and mediates uptake of complement-coated particles
ITGAX	Integrin Alpha X (CD11C)	Receptor for fibrinogen, mediates cell-cell interaction, monocyte adhesion and chemotaxis
LTB	Lymphotoxin beta	Type II membrane protein of TNF family, inducer of inflammatory response
MARCO	Macrophage receptor with collagenous structure	Class A scavenger receptor, pattern recognition receptor that binds gram positive and negative bacteria
MICA	MHC class I polypeptide-related sequence A	Antigen presentation, ligand for NKG2D, mediates cell lysis
MICB	MHC class I polypeptide-related sequence B	Antigen presentation, stress induced self-antigen, ligand for NKG2D, mediates cell lysis
MMP1	Matrix metalloprotease 1	Degrade extracellular matrix, collagenase
MMP12	Matrix metalloprotease 12	Degrade extracellular matrix, Elastin
MMP2	Matrix metalloprotease 2	Degrade extracellular matrix, gelatinase
MMP3	Matrix metalloprotease 3	Degrade extracellular matrix, stromelysin
MMP7	Matrix metalloprotease 7	Degrade extracellular matrix, Matrilysin, involved in wound healing, regulates activity of defensins
MMP9	Matrix metalloprotease 9	Degrade extracellular matrix, gelatinase
MRC1	Mannose receptor C type 1	Endocytosis of glycoproteins, phagocytic receptor for bacteria and fungi
MSR1	Macrophage scavenger receptor 1	Class A scavenger receptor, endocytosis of low density lipoproteins
NFKB1	Nuclear factor of kappa light polypeptide gene enhancer in B-cells 1	Pleiotropic transcription factor, essential immune regulator
PTPRC	Protein tyrosine phosphatase receptor type C (CD45)	Signalling molecule that regulates cell growth, differentiation and mitosis, co-stimulatory molecule required for T cell activation
RAET1E	Retinoic acid early transcript 1E	Ligand for NKG2D, activates NK cells
RAET1G	Retinoic acid early transcript 1G	Ligand for NKG2D, mediates NK cell cytotoxicity
RAET1L	Retinoic acid early transcript 1L	Ligand for NKG2D, antigen binding
RARA	Retinoic acid nuclear receptor alpha	Receptor for retinoic acid
RELA	v-rel avian reticuloendotheliosis viral oncogene homolog A	Subunit of the NFⱪB complex, Pleiotropic transcription factor, essential immune regulator
S100A4	S100 calcium binding protein A4	Regulation of motility and tubulin polymerization
SELL	Selectin L (CD62L)	Cell surface adhesion gene, C-type lectin, rolling of leukocytes on endothelial cells
SERPINA1	Serpin peptidase inhibitor, Clade A	Serine protease inhibitor, primary target is elastase
SERPINB2	Serpin peptidase inhibitor, Clade B	Inhbits urokinase-type plasminogen activator
SIGLEC10	Sialic acid binding Ig-like lectin 10	Member of immunoglobulin superfamily, mediates sialic-acid dependent binding to cells, inhibitory receptor in the immune response
SIRPA	Signal regulatory protein alpha	Immunoglobulin-like cell surface receptor for CD47Mediates negative regulation of phagocytosis, prevents maturation of immature DC
TIMP1	Tissue inhibitor of metalloproteinases 1	Complexes with metalloproteinases and irreversibly inactivates them
TLR2	Toll-like receptor 2	Mediates immune responses to bacterial lipoproteins and other cell wall components
TLR3	Toll-like receptor 3	Mediates immune responses to virus by sensing double stranded RNA
TLR4	Toll-like receptor 4	Mediates immune responses to bacterial lipopolysaccharide
TLR7	Toll-like receptor 7	Mediates immune responses to virus by sensing single stranded RNA
TLR8	Toll-like receptor 8	Mediates immune responses to virus by sensing double- stranded RNA
TLR9	Toll-like receptor 9	Mediates immune responses to bacteria by sensing unmethylated CpG dinucleotides
TNF	Tumor necrosis factor alpha	Proinflammatory cytokine that stimulates cell proliferation, differentiation, apoptosis, cytokine production and causes fever
TNFSF15	Tumor necrosis factor superfamily member 15	Ligand for decoy receptor (DR3) and DR6, activates NFⱪB and MAPK, promotes activation of caspases and apoptosis
TREM1	Triggering receptor expressed on myeloid cells 1	Stimulated neutrophil and monocyte-mediated inflammatory responses, triggers release of proinflammatory chemokines and cytokines
TREM2	Triggering receptor expressed on myeloid cells 2	Triggers actvation of immune responses in macrophages and DCs
ULBP1	UL16 binding protein 1	Ligand for NKG2D, induces production of cytokines and chemokines in primary NK cells
ULBP2	UL16 binding protein 2	Ligand for NKG2D, induces production of cytokines and chemokines in primary NK cells
ULBP3	UL16 binding protein 3	Ligand for NKG2D, induces production of cytokines and chemokines in primary NK cells

Protein function adapted from GeneCards.org

**Table 2:** Relative Log2 transformed gene expression levels of the three subsets and statistical significance the subsets in between.

Gene symbol	Gene expression values in monocyte subsets			T-test values		
	Classical	Intermediate	Non-classical	C vs I	C vs NC	I vs NC
ACTB	16,3	16,9	16,6	1,40E-09	2,40E-03	4,20E-03
ADAM17	5,9	5,8	6,6	9,50E-01	3,00E-01	2,60E-01
BAX	5,2	7,4	6,4	9,90E-04	8,90E-02	1,20E-01
BCL2	2,6	3	2,5	4,80E-01	8,30E-01	3,60E-01
BCL2L1	0,8	2,4	2,1	3,30E-03	1,80E-02	5,90E-01
C1QA	0,1	2,7	3,1	3,80E-07	5,20E-09	5,90E-01
CCR1	3,6	3,1	1,2	3,60E-01	1,70E-05	4,70E-04
CCR2	1,4	0,4	0	9,50E-03	9,40E-05	4,60E-02
CCR5	0	1	0	1,80E-03		2,30E-03
CCR7	0	0	0			
CCR9	0,4	0,1	0,1	1,70E-03	1,70E-02	3,90E-01
CD14	11,7	7,3	4,6	8,80E-17	3,40E-32	7,70E-05
CD163	5,4	1,3	0	1,30E-09	8,30E-19	2,40E-04
CD209	1,1	0,7	0,4	1,20E-01	3,80E-03	2,10E-01
CD33	9,3	8	5,5	1,70E-02	1,50E-09	1,20E-04
CD36	10,3	7,1	1	1,60E-06	1,90E-45	1,00E-18
CD40	6	7,4	5	4,00E-02	1,50E-01	5,30E-04
CD68	14,2	14,9	13,8	4,90E-10	2,40E-01	1,70E-03
CD74	14,9	16,2	14,6	1,30E-16	2,80E-02	2,80E-23
CD93	6,8	5	2,3	5,60E-03	8,10E-12	3,70E-05
CLEC4E	2,2	0,3	0	5,40E-05	1,30E-06	9,70E-02
CSF1R	5,4	10,2	8,9	2,80E-13	3,70E-07	9,70E-03
CST3	13,4	14,3	12,7	3,60E-17	1,60E-02	1,60E-08
CTSB	11,5	12,4	11,1	4,00E-02	5,00E-01	1,80E-03
CTSK	2,5	2,4	1,7	9,60E-01	1,60E-01	1,70E-01
CTSL	0,3	5,2	3,7	1,20E-13	4,80E-09	6,30E-02
CTSS	14,7	14,3	13,8	2,60E-05	1,50E-08	5,00E-04
CX3CR1	5,7	5,1	5	1,90E-01	1,20E-01	7,80E-01
FCGR3A	8,1	11,9	12,3	1,00E-13	3,60E-20	1,50E-01
HLA-DRA	13,2	14,7	12,4	4,00E-15	2,00E-02	1,70E-11
HLA-DRB1	5,4	0,9	2	1,00E-09	2,00E-05	5,30E-02
HMOX1	8,9	12,2	12	3,60E-10	5,00E-08	4,60E-01
IL10	2,4	1,2	0,1	2,30E-02	1,50E-06	1,40E-03
IL12A	5,8	7,6	7	1,60E-03	3,80E-02	3,40E-01
IL15	2,2	2,5	2,9	6,60E-01	2,70E-01	5,00E-01
IL18	2,7	2,9	0,9	7,50E-01	1,80E-03	4,40E-04
IL1B	1,7	3,3	1,1	3,40E-02	2,60E-01	1,80E-03
IL23A	3,3	3,9	3,9	2,20E-01	1,60E-01	1,00E+00
IL6	0	0,1	0,3	3,10E-01	7,50E-02	3,90E-01
IRF4	0,1	0,6	0,6	7,40E-02	1,30E-01	9,10E-01
IRF5	7,1	9,9	8,6	4,20E-07	1,10E-02	6,90E-03
IRF8	6,8	8,1	2,9	6,50E-02	5,20E-08	8,30E-15
ITGAE	0,1	0,3	0,1	4,10E-01	6,20E-01	1,90E-01
ITGAL	7,6	11,4	12	1,70E-10	3,40E-15	2,50E-02
ITGAM	9,8	8,5	2,6	5,20E-02	1,70E-23	1,60E-15
ITGAX	10,5	12,2	11,6	4,90E-09	1,30E-03	6,40E-03
LTB	0,5	2,8	3,4	9,80E-07	7,60E-09	3,50E-01
MARCO	4,3	6,7	2,2	1,30E-03	2,30E-03	2,40E-10
MICA	0,8	0,5	0,8	5,70E-01	9,60E-01	5,20E-01
MICB	3,1	4,7	3,7	1,80E-02	3,40E-01	1,70E-01
MMP1	7,3	7,3	7,5	9,90E-01	7,60E-01	7,80E-01
MMP12	0	0	0	9,20E-01	7,40E-01	8,10E-01
MMP2	0,3	0,7	0,5	8,90E-02	3,20E-01	4,50E-01
MMP3	3,8	6,1	5,7	7,30E-05	7,50E-04	6,10E-01
MMP7	2,5	3,4	2,5	1,40E-02	8,70E-01	9,20E-03
MMP9	0,1	0	0,1	3,20E-01	9,50E-01	3,10E-01
MRC1	0	0	0			
MSR1	0,8	3,7	4,4	2,90E-06	1,00E-08	3,60E-01
NFKB1	4,2	5,8	4,1	4,10E-02	9,10E-01	2,90E-02
PTPRC	8,8	11	10	5,40E-34	2,10E-15	1,10E-10
RAET1E	0,1	0,4	0,4	5,20E-05	4,90E-04	4,00E-01
RAET1G	0	0,1	0	1,60E-01		1,60E-01
RAET1L	1,1	2,1	1,5	9,50E-03	3,20E-01	1,40E-01
RARA	11,8	11,8	11,6	8,50E-01	6,20E-01	5,70E-01
RELA	6,4	8,2	5,2	1,70E-02	1,10E-01	4,50E-05
S100A4	13,1	13,8	13,3	1,00E-08	2,70E-01	2,50E-02
SELL	11,9	7,3	2,4	7,90E-15	7,60E-43	4,30E-12
SERPINA1	13,2	13,9	13,1	4,90E-09	7,50E-01	1,40E-02
SERPINB2	0,9	0,1	0	5,00E-03	1,40E-03	3,20E-01
SIGLEC10	4,6	10	10,1	1,50E-15	1,00E-16	9,50E-01
SIRPA	9,4	7,4	4,1	3,20E-04	5,50E-19	1,10E-07
TIMP1	11,7	13	12,2	2,70E-10	1,50E-01	1,20E-02
TLR2	10	9	8,8	1,90E-02	1,40E-02	7,70E-01
TLR3	0	0	0,1		3,10E-01	3,10E-01
TLR4	8,4	8	7,6	4,20E-01	1,50E-01	5,00E-01
TLR7	0,2	1,5	0,5	1,70E-03	3,20E-01	3,10E-02
TLR8	4,7	5,3	5	4,40E-01	6,90E-01	7,10E-01
TLR9	3,8	4,2	4,6	4,30E-01	2,00E-01	6,00E-01
TNF	5,8	8,4	9,8	6,30E-04	8,00E-10	2,90E-02
TNFSF15	5,8	7	6,4	2,40E-05	1,90E-02	4,50E-03
TREM1	11,2	7,8	4,4	1,60E-08	2,40E-18	9,50E-05
TREM2	0,1	0	0	3,20E-01	3,30E-01	
ULBP1	0	0	0			
ULBP2	0	0	0		3,10E-01	3,10E-01
ULBP3	3,8	5,2	5	3,00E-02	8,20E-02	7,00E-01

C = Classical, I = Intermediate, NC = Non-Classical

### Comparison of gene expression investigated by single-cell PCR gene expression analysis versus micro-array

Previous microarray data have shown that genes within diverse cellular and immunologic process are differentially expressed within the three subsets. We therefore wanted to validate our gene expression data generated by Fluidigm (Table 2) with existing array data. We compared our single cell gene-expression data to current expression profiles published by Wong et al. by looking at the general expression pattern since data have not been derived using the same methods. In agreement with the study conducted by Wong et al., the classical monocytes showed the highest expression of CCR1, CCR2, CD14, CD163, CD36, SELL, and SERPINB2 whereas the intermediate showed highest expression of CD40, CD74, HLA-DRA, MARCO and TIMP, and the non-classical showed the highest expression of C1QA, ITGAL, and SIGLEC10. However, our data deviated from Wong et al., by showing the highest expression of CTSL and HMOX1 by the intermediate monocytes and CX3CR1 by the classical monocytes, which were found by Wong et al. to be most highly expressed by the non-classical subset.

### Functional characteristics of the three subsets

To investigate unique characteristics of each monocyte subset, the genes examined were grouped into categories based on functionality (Table 3). The classical monocytes were shown to have the highest expression of CD93, CD209, CLEC4E, which are genes involved in pathogen recognition and phagocytosis. Also genes encoding proteins involved in migration and adhesion such as the chemokine receptors, CCR1, CCR2, CCR9, CX3CR1, ITGAM and SELL were most highly expressed by the classical monocytes. The expression of genes encoding scavenger receptors was mostly expressed by the classical and intermediate monocytes. As shown earlier, the intermediate cells had the highest expression of genes involved in co-stimulation and antigen presentation, but also genes involved in NK cell and CD8 T cell activation, such as MICB, RAET1E, RAET1G, RAET1L and ULBP3 were enriched on intermediate monocytes. Furthermore, we also found that the intermediate monocytes had the highest expression of genes involved in cell differentiation and cell function, e.g. the genes encoding the transcription factors IRF5 and IRF8, the NFⱪB1 and RELA genes involved in heterodimer formation of the central immunologic regulatory transcription factor NFⱪB, and CSF1R where the encoded protein controls cell differentiation. Also, genes encoding the cytokines IL1β, IL12A, IL18 and IL23A, proteases, such as cathepsins and MMPs, and protease inhibitors, CST3, TIMP1 and SERPINA1 were highly expressed by the intermediate monocytes. In contrast, the non-classical monocytes showed the highest expression of TNF and the metalloprotease ADAM17 gene, which are involved in the processing of TNF from the cell surface. Low expression of genes involved in bacterial phagocytosis were found in the non-classical monocyte subsets, thus they had the highest expression of C1QA, a complement component, and the FcγR3A involved in antibody-mediated phagocytosis. Genes encoding the TLR8 and 9 proteins, which are classical, innate pattern recognition receptors (PRR) were also highly expressed by the non-classical monocytes.

**Table 3:** Functional categorization. The data indicate relative Log2 transformed gene expression levels.

Gene symbol	Gene expression in monocyte subsets		
	Classical	Intermediate	Non-classical
**Antigen presentation**			
CD40	6,0	**7,4**	*5*,*0*
CD74	14,9	**16,2**	*14*,*6*
HLA-DRA	13,2	**14,7**	*12*,*4*
HLA-DRB1	**5,4**	*0*,*9*	2,0
**Adhesion/migration**			
CD33	**9,3**	8,0	*5*,*5*
CD93	**6,8**	5,0	*2*,*3*
CCR1	**3,6**	3,1	*1*,*2*
CCR2	**1,4**	0,4	*0*,*0*
CCR5	*0*,*0*	**1,0**	*0*,*0*
CCR9	**0,4**	*0*,*1*	0,1
CX3CR1	**5,7**	5,1	*5*,*0*
ITGAE	0,1	**0,3**	*0*,*1*
ITGAL	*7*,*6*	11,4	**12,0**
ITGAM	**9,8**	8,5	*2*,*6*
ITGAX	*10*,*5*	**12,2**	11,6
SELL	**11,9**	7,3	*2*,*4*
SIGLEC10	*4*,*6*	10,0	**10,1**
**Differentiation and function**			
BAX	*5*,*2*	**7,4**	6,4
BCL2	2,6	**3,0**	*2*,*5*
BCL2L1	*0*,*8*	**2,4**	2,1
CSF1R	*5*,*4*	**10,2**	8,9
IRF4	*0*,*1*	**0,6**	0,6
IRF5	*7*,*1*	**9,9**	8,6
IRF8	6,8	**8,1**	*2*,*9*
NFKB1	4,2	**5,8**	*4*,*1*
PTPRC	*8*,*8*	**11,0**	10,0
RARA	11,8	**11,8**	*11*,*6*
RELA	6,4	**8,2**	*5*,*2*
S100A4	*13*,*1*	**13,8**	13,3
**Scavenger receptors**			
CD36	**10,3**	7,1	*1*,*0*
CD68	14,2	**14,9**	*13*,*8*
CD163	**5,4**	1,3	*0*,*0*
MARCO	4,3	**6,7**	*2*,*2*
MSR1	*0*,*8*	3,7	**4,4**
**Phagocytosis/bacterial clearance**			
CD93 phagocytosis	**6,8**	5,0	*2*,*3*
CD209	**1,1**	0,7	*0*,*4*
CLEC4E	**2,2**	0,3	*0*,*0*
**Innate immune responses**			
ADAM17	5,9	*5*,*8*	**6,6**
C1QA	*0*,*1*	2,7	**3,1**
CD14	**11,7**	7,3	4,6
FCGR3A	*8*,*1*	11,9	**12,3**
HMOX1	*8*,*9*	**12,2**	12,0
LTB	*0*,*5*	2,8	**3,4**
MICA	0,8	*0*,*5*	**0,8**
MICB	*3*,*1*	**4,7**	3,7
RAET1E	*0*,*1*	**0,4**	0,4
RAET1G	*0*,*0*	**0,1**	*0*,*0*
RAET1L	*1*,*1*	**2,1**	1,5
SERPINB2	**0,9**	0,1	*0*,*0*
SIRPA	**9,4**	7,4	*4*,*1*
TLR2	**10,0**	9,0	*8*,*8*
TLR3	*0*,*0*	*0*,*0*	**0,1**
TLR4	**8,4**	8,0	*7*,*6*
TLR7	*0*,*2*	**1,5**	0,5
TLR8	*4*,*7*	**5,3**	5,0
TLR9	*3*,*8*	4,2	**4,6**
TNFSF15	*5*,*8*	**7,0**	6,4
TREM1	**11,2**	7,8	*4*,*4*
TREM2	**0,1**	*0*,*0*	*0*,*0*
ULBP3	*3*,*8*	**5,2**	5,0
**Cytokines**			
IL10	**2,4**	1,2	*0*,*1*
IL12A	*5*,*8*	**7,6**	7,0
IL15	*2*,*2*	2,5	**2,9**
IL18	2,7	**2,9**	*0*,*9*
IL1B	1,7	**3,3**	*1*,*1*
IL23A	*3*,*3*	**3,9**	3,9
IL6	*0*,*0*	0,1	**0,3**
TNF	*5*,*8*	8,4	**9,8**
**Proteases and protease inhibitors**			
CST3	13,4	**14,3**	*12*,*7*
CTSB	11,5	**12,4**	*11*,*1*
CTSK	**2,5**	2,4	*1*,*7*
CTSL	*0*,*3*	**5,2**	3,7
CTSS	**14,7**	14,3	13,8
MMP1	*7*,*3*	7,3	**7,5**
MMP2	*0*,*3*	**0,7**	0,5
MMP3	*3*,*8*	**6,1**	5,7
MMP7	2,5	**3,4**	*2*,*5*
MMP9	0,1	*0*,*0*	**0,1**
TIMP1	*11*,*7*	**13,0**	12,2
SERPINA1	13,2	**13,9**	*13*,*1*

**Bold** = highest gene expression, Standard = medium gene expression, *Italic* = lowest gene expression among the three monocyte subsets

### Detection of inter-cellular variation

**Fig 2:**
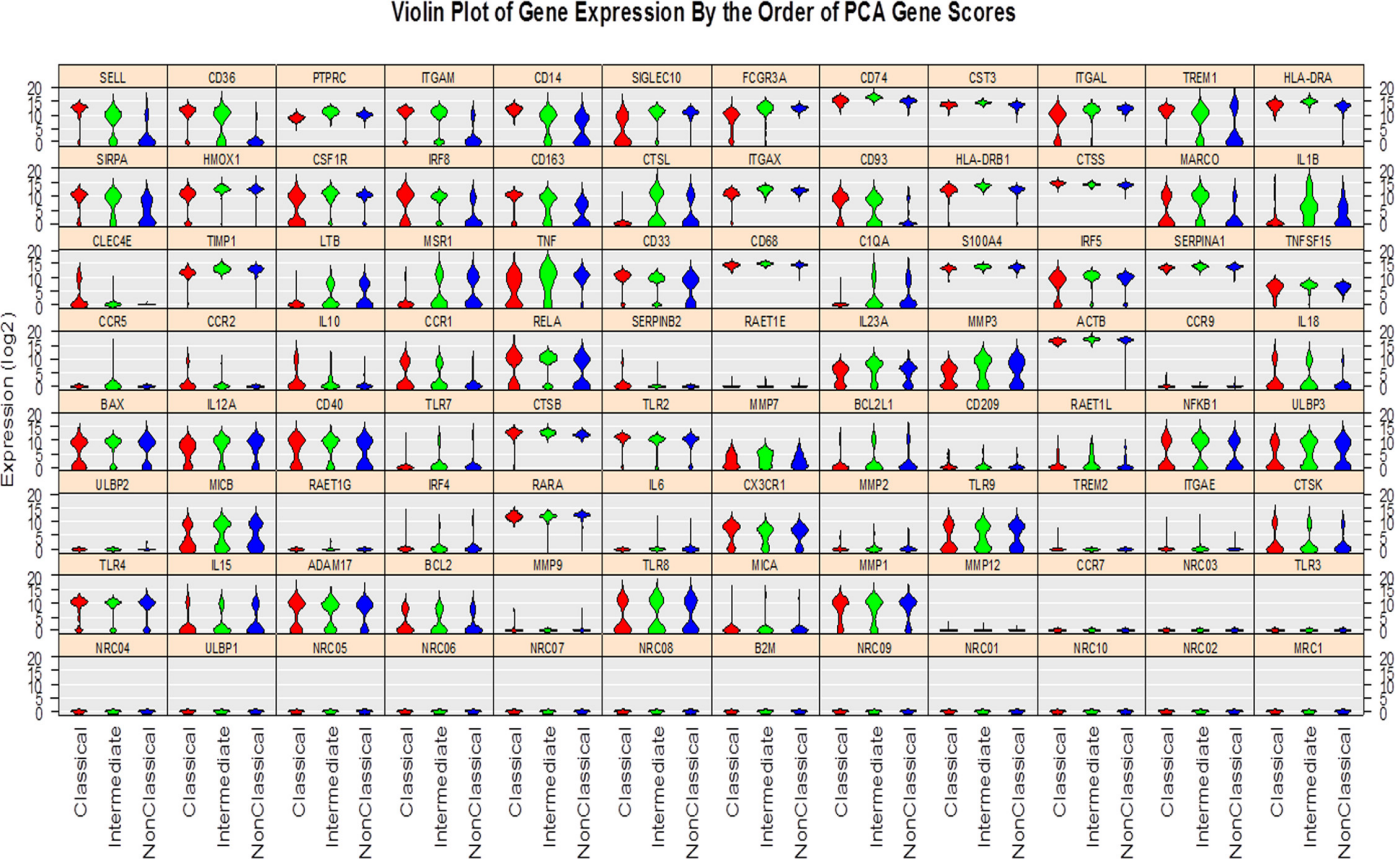
Multimodal variation in expression levels across the three monocyte subsets. Violin plot demonstrating multimodal variation in gene expression levels of the 85 genes examined in the monocyte subsets. The classical monocytes, intermediate monocytes and non-classical monocytes are indicated in the figure by red, green and blue, respectively. The data depict the multimodal expression levels of the genes listed in Table 4 calculated by using the Hartigans dip test (*P <* 0.05).

In contrast to previous gene expression profiling studies [9,11,15], the aim of the current study was to investigate the biological genetic variability within the monocyte subsets by using the Fluidigm single-cell gene expression tool. The probes used for the PCR amplification have been tested with DNA, and were able to amplify and detect transcripts. This technique is therefore able to assess selected genes within the individual cells, and investigate possible multimodal gene expression within cell populations. Using a Hartigans dip test, we identified genes within the three subsets that had multimodal expression (Table 4, and Fig 2). Within the classical, intermediate and non-classical subsets, 37, 39 and 36 genes, respectively showed multimodal expression (Table 4). Some of the genes showed a general multimodal expression in all three subsets such as the co-stimulatory molecule CD40, the scavenger receptor MARCO, the adhesion molecules CD33, CD93 and CX3CR1, the apoptosis genes BAX and BCL2, the genes encoding the cytokines IL12A, IL15 and IL23A, TLR8 and TLR9 and the proteases, CTSK, MMP1 and MMP3. Apart from the genes with multimodal expression in all three subsets, the classical subset showed multimodal expression of the phagocytosis-associated gene CLEC4E, the scavenger receptor gene CD163 and the FcγR3A gene, adhesion and migration genes CCR1, CCR2, ITGAL, and SICLEG10, the cytokines IL10, IL18 and TNF, and genes involved in differentiation such as the transcription factor IRF5. The intermediate monocytes showed generally multimodal expression of the genes involved in apoptosis and adhesion such as BCL2L1 and SELL, respectively, and the scavenger receptor genes CD36, MARCO and MSR1. Furthermore, it was in particular genes involved in regulating immune responses such as C1QA, CD14, IL1β, IL18, TNF, TLR7, TREM1, and SIRPα, and genes encoding the proteases, CTSL and MMP7 that showed multimodal expression. The non-classical monocytes exhibited, in addition to the genes which also showed multimodal expression by the classical and intermediate monocytes, multimodal expression of genes involved in apoptosis, differentiation and activation such as BCL2L1, CSF1R, and IRF5. Similar to the intermediate subset, the non-classical monocytes also showed multimodal expression in a range of immune responsive genes such as ADAM17, C1QA, CD14, LTB, TREM and SIRPα as well as the genes encoding the proteases, CTSK, CTSL, MMP1 and MMP3.

**Table 4:** Multimodal expression in the three monocyte subsets.

G.ene symbol	Gene expression modality			Gene symbol	Gene expression modality		
	Classical	Intermediate	Non-Classical		Classical	Intermediate	Non-Classical
ACTB	*0*,*86*	*0*,*68*	*0*,*97*	IRF8	**0,00**	**0,00**	**0,00**
ADAM17	**0,00**	**0,00**	**0,00**	ITGAE	*1*,*00*	*1*,*00*	*1*,*00*
BAX	**0,00**	**0,00**	**0,00**	ITGAL	**0,00**	*0*,*83*	*0*,*95*
BCL2	**0,00**	**0,00**	**0,00**	ITGAM	**0,00**	**0,00**	**0,00**
BCL2L1	*0*,*94*	**0,00**	**0,00**	ITGAX	*0*,*99*	*0*,*86*	*0*,*95*
C1QA	*1*,*00*	**0,00**	**0,00**	LTB	*0*,*99*	**0,00**	**0,00**
CCR1	**0,00**	**0,00**	*0*,*31*	MARCO	**0,00**	**0,00**	**0,00**
CCR2	**0,03**	*1*,*00*	*1*,*00*	MICA	*0*,*98*	*1*,*00*	*0*,*99*
CCR5	*1*,*00*	*0*,*99*	*1*,*00*	MICB	**0,00**	**0,00**	**0,00**
CCR9	*1*,*00*	*1*,*00*	*1*,*00*	MMP1	**0,00**	**0,00**	**0,00**
CD14	*0*,*86*	**0,00**	**0,00**	MMP2	*1*,*00*	*1*,*00*	*0*,*99*
CD163	**0,00**	*0*,*15*	*1*,*00*	MMP3	**0,00**	**0,00**	**0,00**
CD209	*0*,*94*	*0*,*99*	*1*,*00*	MMP7	*0*,*34*	**0,03**	*0*,*22*
CD33	**0,02**	**0,00**	**0,00**	MMP9	*1*,*00*	*1*,*00*	*1*,*00*
CD36	*0*,*11*	**0,00**	*0*,*49*	MSR1	*0*,*92*	**0,00**	**0,00**
CD40	**0,00**	**0,00**	**0,00**	NFKB1	**0,00**	**0,00**	**0,00**
CD68	*0*,*70*	*0*,*99*	*0*,*99*	PTPRC	*0*,*96*	*0*,*72*	*0*,*95*
CD74	*0*,*76*	*0*,*95*	*0*,*98*	RAET1E	*1*,*00*	*0*,*97*	*0*,*99*
CD93	**0,00**	**0,00**	**0,00**	RAET1G	*1*,*00*	*1*,*00*	*1*,*00*
CLEC4E	**0,00**	*1*,*00*	*1*,*00*	RAET1L	*0*,*86*	*0*,*12*	*0*,*67*
CSF1R	**0,00**	*0*,*94*	**0,00**	RARA	*0*,*98*	*1*,*00*	*0*,*83*
CST3	*0*,*52*	*0*,*62*	*0*,*88*	RELA	**0,00**	**0,00**	**0,00**
CTSB	*0*,*25*	*0*,*75*	*0*,*70*	S100A4	*0*,*82*	*0*,*95*	*0*,*91*
CTSK	**0,00**	**0,00**	**0,00**	SELL	*0*,*97*	**0,00**	**0,00**
CTSL	*1*,*00*	**0,00**	**0,00**	SERPINA1	*0*,*99*	*0*,*96*	*0*,*99*
CTSS	*0*,*85*	*0*,*99*	*0*,*99*	SERPINB2	*0*,*34*	*1*,*00*	*1*,*00*
CX3CR1	**0,00**	**0,00**	**0,00**	SIGLEC10	**0,00**	*0*,*10*	*0*,*70*
FCGR3A	**0,05**	*0*,*96*	*0*,*95*	SIRPA	*0*,*25*	**0,00**	**0,01**
HLA.DRA	*0*,*90*	*0*,*94*	*0*,*61*	TIMP1	*0*,*96*	*0*,*93*	*0*,*90*
HLA.DRB1	**0,00**	*0*,*92*	**0,02**	TLR2	*0*,*85*	*0*,*72*	**0,04**
HMOX1	**0,00**	*0*,*84*	*0*,*98*	TLR3	*1*,*00*	*1*,*00*	*1*,*00*
IL10	**0,00**	*0*,*49*	*1*,*00*	TLR4	**0,00**	**0,00**	**0,00**
IL12A	**0,00**	**0,00**	**0,00**	TLR7	*1*,*00*	**0,02**	*1*,*00*
IL15	**0,00**	**0,00**	**0,00**	TLR8	**0,00**	**0,00**	**0,00**
IL18	**0,00**	**0,00**	*0*,*24*	TLR9	**0,00**	**0,00**	**0,00**
IL1B	*0*,*07*	**0,00**	*0*,*67*	TNF	**0,00**	**0,00**	*0*,*70*
IL23A	**0,00**	**0,00**	**0,00**	TNFSF15	*0*,*83*	*0*,*80*	*0*,*75*
IL6	*1*,*00*	*1*,*00*	*1*,*00*	TREM1	*0*,*66*	**0,00**	**0,00**
IRF4	*1*,*00*	*0*,*98*	*0*,*99*	TREM2	*1*,*00*	*1*,*00*	*1*,*00*
IRF5	**0,00**	*0*,*72*	**0,00**	ULBP3	**0,00**	**0,00**	**0,00**

**Bold** = Multimodal expression *P* <0.05, *Italic* = Unimodal expression

We demonstrate here that single-cell gene-expression analysis is a valuable tool in detecting multi-modality within cell populations. To exclude that the bi-modality we observed is not caused by a so-called “drop out effect” due to technology artefact, we calculated the relationship between log-transformed transcript expression data and Hartigan’s dip test p-value. When we include the cells with an expression we found no difference comparing uni- and bi-modal genes (p = 0.54). If genes with low expression were more prone to drop out effects we would have expected an overrepresentation of these amongst the bi-modal genes.

### Classification of subpopulations

**Fig 3:**
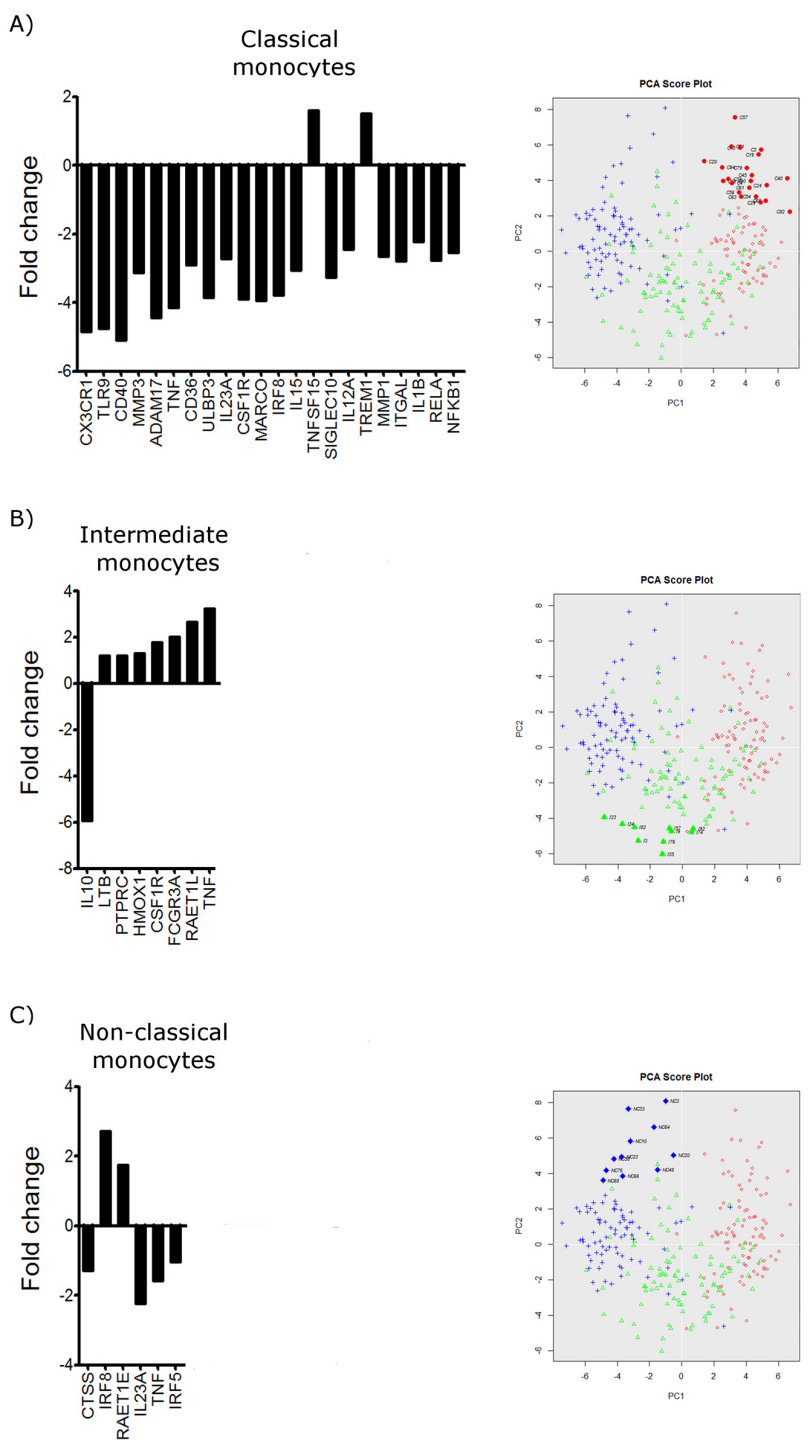
Expression level of genes deviating in identified subgroups. Subgroups of cells were identified based on the PCA of single-cell PCR gene expression analysis data. One subgroup among the classical monocytes, intermediate monocytes and non-classical monocytes, and co-expression of genes within the subgroups were assessed using a student T test (*P <* 0.05). A) Bar graph demonstrating the differentially expressed genes by the subgroup within the classical monocyte subset identified on the PCA score plot. The subgroup is marked by filled red circles in the PCA score plot. B) Bar graph demonstrating the differentially expressed genes by the subgroup within the intermediate monocyte subset identified on the PCA score plot. The subgroup is marked by filled green triangles in the PCA score plot. C) Bar graph demonstrating the differentially expressed genes by the subgroup within the non-classical monocyte subset identified on the PCA score plot. The subgroup is marked by filled blue pluses in the PCA score plot.

With single-cell gene expression profile we are able to study the heterogeneity between and within cell populations. In addition to the multimodal analysis we also analysed co-expression of genes within the classical monocytes, intermediate monocytes and non-classical monocytes in order investigate potential subgroups with distinct gene expression profiles. Using the PCA of single-cell PCR gene expression analysis data it was possible to identify one subgroup of monocytes that diverged from the main populations (Fig 3 and S1 Table). Within the population of classical monocytes, we found one subgroup that showed differential expression of 22 genes (Fig 3A). Of these genes, only 2 genes showed a higher expression than the main population, namely TNFSF15 and TREM1. The remaining genes were all found to have lower expression than in the main population. Within the intermediate subset, we also identified a subgroup of cells displaying differential gene expression of eight genes (Fig 3B). Compared to the main population of intermediate monocytes, only IL10 was found to have lower expression whereas LTB, PTPRC, HMOX1, CSF1R, FCGR3A, RAETL1 and TNF were all found to be significantly more highly expressed. Likewise, within the non-classical monocytes we found a subgroup of cells with a co-expression of IRF8 and RAET1E at higher levels than the main population but showing lower gene expression of CTSS, IL123A, IRF5 and TNF (Fig 3C).

Comparing the expression of genes within the subgroup of cells identified by the PCA plot, we demonstrate that the classical, intermediate and non-classical human blood monocytes each contain a subgroup of cells characterized by distinct gene signatures. This highlights the great diversity and possible plasticity within the human monocyte sub-populations.

## Discussion

Comprehensive genome-wide analyses have shown distinct heterogeneity within human monocytes [9,11,15]. Three subsets have been identified in humans, namely the classical, intermediate and non-classical monocytes. In this study we have used the single-cell PCR gene analysis technique from Fluidigm, and confirmed existing data defining three monocyte subsets and demonstrated differential gene expression among the classical, intermediate and non-classical monocytes. Moreover, the differential expression of genes encoding cell surface molecules identified, for instance, the expression of CD163 and TREM2 in classical monocytes and the expression of CCR5 and RAET1G in intermediate monocytes, could be useful when discriminating the three monocyte subsets by methods such as flow cytometry. Furthermore, we have shown inter-cellular variation of genes within each subset, which highlights the heterogeneity of monocytes as a diverse group of innate immune leukocytes containing possible further functional subclasses. Thus, by our study, performed with one donor, we here demonstrate the possibilities of subgrouping the monocytes using single-cell PCR gene expression analysis.

The single-cell gene expression analysis presented here demonstrates high phagocytic capacity (CD93, CD209, CLEC4E, and SIRPA) of the classical monocytes, which is in agreement with previous studies. Also, a high expression of a broad range of innate sensing receptor genes, pro-inflammatory genes and genes linked to innate immune responses (CD14, TLR2, TLR4 and TREM1) are observed for the classical monocytes [9,11,15]. In addition to these findings we have previously shown that the classical monocytes secrete high levels of IL-1β, IL-10, and TNFα, and that most IL-6 and MMP1 is produced in response to LPS and immune-complex activation by the classical subset compared to the intermediate and non-classical subset [12]. In accordance to earlier micro-array studies, we also saw the highest expression of genes involved in migration. These findings, together with our previous results showing the highest migratory capacity of the classical monocytes towards CCL2 [12] may underline the capacity of classical monocytes to support inflammation and mount an immune response towards microbial pathogens.

The intermediate monocytes had high antigen presenting potential, which has also been demonstrated by their property to induce CD4^+^ T cell proliferation [15]. Moreover, the intermediate monocytes were the only subset that expressed CCR5, a chemokine receptor responsible for recruiting dendritic cell (DC) precursors from blood to the draining lymph nodes [17]. In addition, our data demonstrate higher gene expression of several cytokines (IL1β, IL12A, IL18, and IL23A) which are important in inducing functionally distinct CD4^+^ T helper (Th) cells. IL12 plays a role in the differentiation of Th1 cells, whereas IL6 and IL23 are important in driving and sustaining the differentiation of Th17 cells [18]. Moreover, IL12, IL15, IL18 and TNFSF15 are involved in the induction of T cell receptor-independent cytokine production by CD4^+^ T cells [19–21]. The intermediate monocytes also showed a higher expression of genes linked to the activation status such as apoptosis regulation (BAX and BCL2), cell differentiation and regulation (CSF1R, IRF5, IRF8, NFⱪB1, RELA and PTPRC). This may suggest that these cells are more activated than the classical monocytes.

The CD16^+^ monocytes have been shown to adhere to the endothelium and mediate arrest through the CX3CL1-CX1CR3 interaction [22]. Additionally, the non-classical monocytes are thought to patrol the vasculature and selectively respond to viral infected- or damaged cells. In line with previous data, in non-classical monocytes we found considerably higher expression of genes coupled to complement (C1QA) and FcR-mediated phagocytosis (FcRγ3A), adhesion (ITGAL and SIGLEC10) and TLR9. However, we find that the genes encoding TLR7 and TLR8, which sense nucleic acids and viruses, are mostly expressed by the intermediate subset. Moreover, we find the highest expression of the CX3CR1 gene in the classical subset, albeit with small variation among the three subsets. This latter finding conflicts with previous data showing high cell surface expression of CX3CR1 on non-classical monocytes and their capacity to respond to damaged cells and viral infections. However, these observations in gene expression do not always correspond to the actual protein expression. Thus difficulty in functional interpretation of genes is a limitation to transcriptome analysis and may explain the discrepancies between observed protein expression and gene analysis data.

The advantage of single-cell gene expression analysis compared to micro-array is that it provides the possibility to analyse gene expression within single cells, facilitating the discovery of multimodal expression on single cells and possible new subpopulations in cell populations identified to date [23,24]. Therefore, in addition to comparing our results with previous micro-array data, we also investigated the intercellular variation among the monocyte subtypes and analysed data for co-expression of genes within each monocyte subset. Interestingly, we could demonstrate that multimodal gene expression is present in all three subsets. The transcription factors IRF8, NFⱪB1 and RELA are among the genes that show multimodal expression in all three subsets. Also genes involved in apoptosis regulation (BAX and BCL2) and cell adhesion (CD33, CD93, CX3CR1 and ITGAM) are expressed multimodally in all three subsets. This multimodality seen in the monocyte subsets may be a reflection of differential maturation and immune activation status. In addition, the classical monocytes also show multimodal expression of the chemokine receptor genes (CCR1 and CCR2), adhesion genes (ITGAL and ITGAM) and innate immune response genes (TLR4, TLR8-9, IL10, IL12A, IL15, IL18, and IL23A), favouring evidence for potentially immune activated cells. However, further data are needed to establish if this is in fact the case, thus these potentially different activation states could be of importance in light of the monocytes ability to extravasate into tissues and respond to pathogenic stimulation. For example, cells expression high levels of CCR2 together with CLEC4E, TLR4, and TNF-a might be more prone for migration and response to bacteria, in contrast to monocytes expressing low levels of CCR2. Also, cells expressing high levels of FCGR3A, CD36 and CD163 might be more prone for scavenging and phagocytosis. Several genes encoding immune responses also show multimodal expression within the intermediate subset together with genes encoding the proteases. Though our study has been carried out using monocytes from a healthy donor, the data presented here serve as a basis for discovering targets on certain subpopulations of cells that may be implicated in disease pathogenesis. In addition, single-cell PCR analyses have revealed that colon cancer tissues contain cell populations distinct from healthy colon, which were not identified by immunohistochemistry or flow cytometry [25]. Moreover we identified a subgroup of cells within the classical group of monocytes that may be less activated given lower expression of, for example genes within the NFⱪB complex, IRF8, and genes encoding several cytokines such as IL1b, IL12A, IL15, IL23A and TNF. In contrast, we describe here a subgroup of cells among the intermediate monocytes, showing higher gene expression of TNF and FCGR3A and lower expression of IL10, suggesting that the cells of this subgroup are more differentiated towards an immune-activated phenotype. The transcription factor IRF8 is known to be involved in the differentiation of monocytes and DCs [26–28]. The subgroup identified in the non-classical cells showed higher gene expression of IRF8 and lower gene expression of IRF5, IL23A and TNF. This could imply that this subgroup of cells is more differentiated towards dendritic cells. Of note, non-classical monocytes are likewise shown to have a higher propensity to become dendritic cells [29].

The advantage of using single-cell gene expression profiling methods compared to micro-array is the ability to distinguish potential target cells or subgroups of cells implicated in disease pathogenesis. However, the limitation of the single-cell gene expression technique is its being dependent on a predesigned panel of primers compared to the broad dataset obtained using micro-array, which is not limited to investigating predesigned genes only.

## Materials and Methods

### Monocyte Purification

A buffy coat from a healthy donor was purchased from the Clinical Immunology Blood Bank, The State University Hospital, Copenhagen. Peripheral Blood Mononuclear Cells (PBMC) were obtained using Ficoll-paque Plus (GE-Healthcare Bio-sciences AB, Uppsala, SE) density centrifugation. Untouched monocytes were isolated from PBMC by negative selection using antibody-coated magnetic bead separation (EasySep Human Monocyte Enrichment Kit without CD16 depletion, Stemcell Technologies, Vancouver, Canada) according to the manufacturers’ instructions. The study was approved by the Regional Ethics Committee in Lund, Sweden.

### FACS

Monocytes and LPC were subsequently washed in PBS containing 2% fetal calf serum (FCS) (all from Gibco, Paisley, UK) prior to staining for 15 minutes at 4°C in the dark. The following antibodies were used for surface staining: APC-conjugated anti-CD3 (UCHT1), Pacific blue-conjugated anti-CD14 (M5E2), PerCP-conjugated anti-CD45 (2D1), FITC-conjugated anti-CD64 (10.1), PE-conjugated anti-HLA-DR (TU36) (all from BD Pharmingen), PerCP-conjugated anti-CD16 (3G8), Live/Dead Fixable Near-IR (all from Invitrogen Molecular Probes). The three monocyte subpopulations, classical (CD14++CD16-), intermediate (CD14++CD16+) and non-classical (CD14+CD16++) were single-cell sorted according to the expression of CD14 and CD16. Each cell population was sorted into a 96-well plate using fluorescence-activated cell sorting (FACS) on a BD FACS ARIAII.

### Single-cell gene expression analysis

The monocytes were sorted into 96-well plates by FACS with the target of one cell in each well containing 5 μl RT Mix solution (mixture of VILO reaction mix, SUPERase-ln and 10% NP40 according to the manufacturer’s protocol). Samples were frozen on dry ice and after thawing, synthesis of cDNA was performed with SuperScript VILO (Invitrogen). Specific targets amplification (STA) was done with a mixture of 85 PCR primers (see Table 1) using Taqman preamp master mix (Invitrogen) running 22 cycles. The probes used have all been tested with DNA and were able to amplify and detect transcripts. Residual primers were subsequently removed by treatment with Exonuclease I (New England Biolabs).

After the clean-up step, 6 μl of STA PCR product from each sample was transferred to a new microtiter plate and a standard qPCR (TaqMan 2x Universal PCR Master Mix, Applied Biosystems) with a Taqman assay (Invitrogen) directed against 18S, was performed to identify empty wells. Wells with no 18S Ct value or with an 18S Ct value above 40 were considered empty.

Single-cell qPCR was performed on a Fluidigm BiomarkHD instrument using SSO Fast EvaGreen SUpermix (Bio-Ras Laboratories) according to the manufacturer’s protocol. All primer pairs from genes listed in Table 1 were used. The data were analysed using the SINGuLAR R package (Fluidigm) and expression values were normalised to that of ACTB.

Sub-groups for each monocyte sub-type were defined from the initial principal component analysis (PCA) plot (Fig 1B). Differentially regulated genes in each sub-group were identified by comparing gene expression in the subgroup versus the rest of the cells in the sub-type using a Students t-test. Differentially regulated genes were defined as those with a p-value < 0.05 and a log2 fold change of at least 1.

## Supporting Information

S1 Table List of genes differentially expressed by the monocyte subgroups. (DOCX)
