# Supplementary material for: A Single-Cell Gene-Expression Profile Reveals Inter-Cellular Heterogeneity within Human Monocyte Subsets
Source: PLoS One. 2015 Dec 9;10(12):e0144351. doi: 10.1371/journal.pone.0144351 (PMC4674153; doi:10.1371/journal.pone.0144351)
Supplement: S1 Table — (DOCX) [file pone.0144351.s001.docx]

S1 Table. List of genes differentially expressed by the monocyte subgroups

Gene symbol P-value Fold Change

| **Classical** |  |  |
| --- | --- | --- |
| CX3CR1 | 1,10195E-08 | -4,829161404 |
| TLR9 | 8,27702E-06 | -4,738396737 |
| CD40 | 9,70853E-06 | -5,077699566 |
| MMP3 | 0,000170284 | -3,122591439 |
| ADAM17 | 0,00039325 | -4,416410414 |
| TNF | 0,000694727 | -4,128849267 |
| CD36 | 0,000742008 | -2,875939319 |
| ULBP3 | 0,000755252 | -3,846702142 |
| IL23A | 0,001145056 | -2,697780428 |
| CSF1R | 0,001383555 | -3,892064254 |
| MARCO | 0,001888722 | -3,934024147 |
| IRF8 | 0,002054832 | -3,773312996 |
| IL15 | 0,004222286 | -3,038289596 |
| TNFSF15 | 0,006442843 | 1,59100755 |
| SIGLEC10 | 0,007812757 | -3,257590281 |
| IL12A | 0,008995716 | -2,422414665 |
| TREM1 | 0,015659392 | 1,504514177 |
| MMP1 | 0,018465758 | -2,630275777 |
| ITGAL | 0,023875777 | -2,771927834 |
| IL1B | 0,026549087 | -2,207808673 |
| RELA | 0,039020522 | -2,753807226 |
| NFKB1 | 0,047433133 | -2,515691878 |
| **Intermediate** |  |  |
| IL10 | 0,045828445 | -5,91362543 |
| LTB | 0,043905753 | 1,179327252 |
| PTPRC | 0,000667036 | 1,198177222 |
| HMOX1 | 5,38402E-05 | 1,28654385 |
| CSF1R | 0,00019443 | 1,752748834 |
| FCGR3A | 0,004974911 | 1,990606941 |
| RAET1L | 0,022233522 | 2,653346695 |
| TNF | 0,00590914 | 3,209075004 |
| **Non-classical** |  |  |
| CTSS | 2,90953E-05 | -1,27380509 |
| IRF8 | 0,001702771 | 2,710897383 |
| RAET1E | 0,008145836 | 1,738993438 |
| IL23A | 0,014827907 | -2,22496423 |
| TNF | 0,022148335 | -1,560129844 |
| IRF5 | 0,027975293 | -1,012371831 |
